# Capturing cognitive health disparities on the national and state level using 2023 proposed federal racial and ethnic categories: the case for a Middle Eastern and North African checkbox

**DOI:** 10.1002/alz.090540

**Published:** 2025-01-09

**Authors:** Tiffany B Kindratt, Alexandra Smith

**Affiliations:** ^1^ University of Texas at Arlington, Arlington, TX USA

## Abstract

**Background:**

Most research on cognitive difficulties in the US focus on minoritized groups that are part of the federal minimum reporting guidelines, include White, Black, Hispanic/Latino, Asian, American Indian/Alaskan Native (AI/AN), and Native Hawaiian/Other Pacific Islander (NH/OPI) individuals. Research has begun to describe cognitive difficulties among Middle Eastern and North African (MENA) adults, but it is limited because they are defined as White. In 2023, the Office of Management and Budget (OMB) proposed adding a separate checkbox for MENA individuals. Our objective was to estimate and compare the odds of cognitive difficulties among MENA adults compared to White, Black, Hispanic/Latino, Asian, AI/AN, NH/OPI adults in the US and four states (California, New York, Michigan, and Texas) after adjusting for sociodemographic characteristics.

**Method:**

We analyzed data from the 2017‐2021 American Community Survey (ages≥45 years; n = 7,284,988). Cognitive difficulties (yes/no) among adults (ages 45+ years) who self‐identified their race/ethnicity from one of the six minimum reporting categories (White, Black, Hispanic/Latino, AI/AN, NH/OPI) were compared to those who reported a MENA ancestry/birthplace.

**Result:**

MENA adults in the US had greater odds of reporting cognitive difficulties compared to White, Black, Hispanic, Asian and AI/AN adults, but there was no difference when compared to NH/OPI adults after adjusting for covariates. In California, MENA adults had higher odds of cognitive difficulties compared to White, Black, Hispanic/Latino, Asian, and NH/OPI adults. In New York, MENA adults had higher odds of cognitive difficulties compared to Asian adults. MENA adults in New York had lower odds of cognitive difficulties when compared to Black, Hispanic/Latino, and AI/AN adults. In Michigan, MENA adults had lower odds of cognitive difficulties than Black, but higher odds than Hispanic/Latino and Asian adults. In Texas, MENA adults had higher cognitive difficulties compared to White, Hispanic/Latino, and Asian adults.

**Conclusion:**

This study expands on previous research uncovering cognitive difficulties among Arab Americans using the 2023 OMB proposal to include MENA as a checkbox as part of the minimum reporting standards for race/ethnicity in the US. It also provides the first state‐based estimates of cognitive difficulties among MENA adults living in California, New York, Michigan, and Texas.

